# Effect of artificial intelligence driven therapeutic lifestyle changes (AI-TLC) intervention on health behavior and health among obesity pregnant women in China: a randomized controlled trial protocol

**DOI:** 10.3389/fpubh.2025.1580060

**Published:** 2025-05-12

**Authors:** Xiaoyun Wang, Yang Bai, Wenzhuo Li, Chenxin Yang, Linjing Zhang, Hongyi Zhu, Rantong Bao, Yang Jiang, Fei Wang, Huanfang Wang

**Affiliations:** ^1^Pediatric Department, Inner Mongolia Maternal and Child Health Care Hospital, Hohhot, China; ^2^School of School of Public Administration and Policy, Renmin University of China, Beijing, China; ^3^Medical College, Qingdao University, Qingdao, Shandong, China; ^4^School of Health Management, China Medical University, Shenyang, China; ^5^Xiang Ya Nursing School, Central South University, Changsha, Hunan, China; ^6^School of Nursing, Tianjin Medical University, Tianjin, China; ^7^Department of Quality Management, Affiliated Hospital of Inner Mongolia Medical University, Hohhot, China; ^8^Jitang College, North China University of Science and Technology, Tangshan, Hebei, China; ^9^Shandong Key Laboratory of Reproductive Research and Birth Defect Prevention, Department of Gynecology, Shandong Provincial Hospital Affiliated to Shandong First Medical University, Jinan, Shandong, China; ^10^Department of Gynecology, Inner Mongolia Maternity and Child Health Care Hospital, Hohhot, China

**Keywords:** obesity, pregnant women, artificial intelligence, therapeutic lifestyle change, randomized controlled trial

## Abstract

**Introduction:**

Obesity has reached epidemic proportions globally, posing significant challenges to public health and economic stability. In China, the prevalence of obesity is increasing rapidly, particularly among pregnant women, who face unique risks due to the complex interplay between obesity and pregnancy outcomes. This study aims to evaluate the effectiveness of an Artificial Intelligence-driven Therapeutic Lifestyle Change (AI-TLC) intervention in improving health behaviors and outcomes among obese pregnant women in China.

**Methods and analysis:**

This randomized controlled trial will recruit pregnant women aged 18 years or older with a singleton pregnancy between 8 and 12 weeks of gestation and a pre-pregnancy BMI of ≥30.0 kg/m^2^. Participants will be randomly assigned to one of three groups: a manual intervention group, an AI intervention group, and a combined AI and manual intervention group. The intervention will focus on therapeutic lifestyle changes, including behavioral modifications, dietary adjustments, and physical activity promotion, supported by a multidisciplinary team. Primary outcomes will include maternal BMI, weight, and adverse pregnancy outcomes, while secondary outcomes will assess physiological indicators, quality of life, mental health, and lifestyle factors.

**Results:**

The study will evaluate the effects of health interventions on obese pregnant women through primary outcomes (e.g., BMI, weight, adverse pregnancy outcomes) and secondary outcomes (e.g., physiological indicators, quality of life, mental health) using various statistical methods. The results will provide insights into the intervention's effectiveness and cost-effectiveness across different socioeconomic groups.

**Discussion:**

The anticipated findings are expected to demonstrate the efficacy of AI-TLC interventions in managing obesity during pregnancy. This study will contribute valuable evidence to the limited research on AI-based interventions for obese pregnant women, offering potential implications for the development of personalized, efficient, and innovative health strategies. The findings may also inform public health initiatives aimed at improving maternal and child health outcomes in the context of obesity.

## 1 Introduction

Obesity is an abnormal or excessive fat accumulation that can impair health. A research study shows that the total number of obese children, adolescents and adults worldwide by 2022 has exceeded 1 billion, of which 879 million are adults. Since 1990, obesity rates among adults have more than doubled among women (1). In China, the problem of overweight and obesity among residents is increasingly prominent, and the rate of overweight and obesity among all age groups in urban and rural residents continues to rise, and more than half of adult residents are overweight or obese. Obesity is not only an independent health problem, but also an important risk factor for multiple chronic diseases such as type 2 diabetes, cardiovascular disease, hypertension, and so on (1, 2). Obesity often coexists with other chronic diseases, creating a complex network that complicates treatment and management. Furthermore, obesity is linked to several biological mechanisms, such as inflammation, oxidative stress, and endocrine disorders, which may collectively contribute to the development of chronic diseases (3). With the increasing rate of obesity worldwide, the incidence of chronic diseases increases, placing a substantial burden on individual health, healthcare systems and socioeconomic (4).

Maternal obesity is associated with a range of adverse pregnancy outcomes, including gestational diabetes mellitus, gestational hypertension, preeclampsia, need for cesarean delivery, and large for gestational age (5–8). These complications not only affect the health of the mother during pregnancy but may also have long-term consequences for the child, such as increased risk of prematurity, large for gestational age, birth-related trauma, hypoglycaemia, neonatal intensive care unit admission, stillbirth, increased risk of obesity and metabolic disorders in later life (8–11).

According to the study, obese patients had 46% more hospitalization, 27% more clinic and outpatient costs and 80% more prescription drugs compared with normal weight individuals (12). In recent years, several guidelines and expert consensus on obesity and chronic diseases have been published at home and abroad, but most of them are suggestions for evaluation methods or diagnosis and treatment procedures, and lack systematic prevention and management countermeasures. Therefore, it is important to study effective strategies for chronic obesity intervention to improve public health.

To address this challenge, comprehensive interventions are needed. At present, foreign interventions for obesity include lifestyle intervention, weight loss drugs and bariatric surgery. Lifestyle changes are fundamental to obesity management, including healthy eating, regular exercise, and psychological support. Most guidelines for health professionals managing patients with obesity recommend integrated lifestyle planning as a first step (13). Other scholars found that within 12 months, lifestyle and behavioral intervention alone resulted in an average weight reduction of intervention group members, and a 46% remission rate of type 2 diabetes (14). Maintaining weight loss is challenging long term through life changes for most people, so clinical practice guidelines recommend multiple ways of treatment, including taking weight loss drugs or performing bariatric surgery. Regulators around the world have approved several weight-loss drugs for long-term weight management: orlistat, phentermine, naltrexone-non-acetone (15). In addition, Martin Bossart and others showed that a combination of glucagon-like peptide-1 (GLP-1), glucose-dependent insulin polypeptide (GIP) and glucagon (GCG) active tripeptide enzyme (SAR441255) to improve weight loss and blood glucose control, while buffer the risk of long-term GCG receptor agonists, the greatest potential in weight loss drugs (1). Common bariatric surgery includes laparoscopic sleeve gastrectomy, gastric bypass bariatric surgery and anastomotic gastric bypass surgery.

In China, traditional Chinese medicine is early on obesity, and has unique advantages in comprehensively improving the symptoms of obese patients, regulating the blood lipid level in the body, and improving the waist and hip circumference of obesity (16).

Drugs and surgery are undoubtedly the quickest options, but their safety profile has yet to be considered. Carolina et al. pointed out that the weight loss response of weight loss drugs varies widely among individuals and their adverse effects are different, such as liststa and Gl-1-1 receptor agonists mainly have adverse effects on the gastrointestinal tract (17). The most common surgical treatment is laparoscopic sleeve gastrectomy as well as gastric bypass bariatric, with long-term results and strong evidence of safety (18). But both procedures still cause complications such as gallstone and malnutrition (15). In this way, lifestyle interventions have their unique advantages in terms of safety and convenience.

Therapeutic Lifestyle Change (TLC) is an intervention to treat and prevent chronic diseases by improving lifestyle. It includes measures such as dietary modification, increased physical activity, smoking cessation, and weight loss, designed to reduce risk factors for disease and improve patients' overall health. The effect of therapeutic lifestyle interventions has been demonstrated in several studies. For example, meta-analysis of RCTs and non-RCTs showed that lifestyle interventions had a borderline effect on restricting gestational weight gain (19). Another study found that TLC interventions could improve criteria for metabolic syndrome and further regulate in combined with medication (such as amlodipine/atorvastatin) (20). However, at present, this technology has hardly been applied to patients with co-patients of obese chronic diseases. Therefore, we would adopt therapeutic lifestyle intervention for co-patients with obese chronic diseases, and study the effect of the intervention and the adaptation of people of all ages.

Although previous studies proved that antenatal dietary and lifestyle intervention in obese pregnant women reduces maternal pregnancy weight gain, how to implement the lifestyle intervention in the most effective and low labor cost way still need to be studied (21). In recent years, AI intervention has attracted attention as a new intervention, and AI-based intervention research is being rapidly developed in China. For example, studies explored the current state of development, protocol guidelines, reporting norms, and challenges facing clinical trials of AI-based interventions (22). Internationally, the research of AI intervention has also made significant progress. For example, a study published in Nature-Medicine explored how AI guidance can help with the early detection of heart disease in routine practice. This study indicated the positive results of low ejection fraction screening through the electronic medical records, and found that the AI intervention overall improved the diagnostic rate of low ejection fraction by 32% (23). This indicates the great potential of the application of AI intervention in the field of medical diagnosis. Despite the remarkable results of AI interventions in some areas, some challenges exist. For example, the development of the CONSORT-AI extension and SPIRIT-AI extension guidelines suggests that interventions involving AI need to be rigorously evaluated prospectively to clarify its health impact (24, 25). Therefore, the development of higher quality AI applications and evaluate their potential to improve the condition of obese chronic disease patients remains an important area that we need to study.

In China, patients with obesity and chronic diseases face severe challenges. Internationally, interventions for patients with obesity and chronic diseases are often supported by professional medical teams and community health programs. In contrast, Chinese patients tend to rely on hospitals and themselves. In view of China, therapeutic lifestyle intervention combined with AI technology, as an innovative intervention, is easy to implement and cost-effective. Through AI technology, personalized lifestyle advice can be provided, monitoring patient health data and timely adjusting treatment options. This intervention not only facilitates patients to receive management at home, but also can deliver professional support to meet the needs of patients in the process of lifestyle change effectively and efficiently. Therefore, research targeting this area has important practical significance and urgency.

This study designed a randomized controlled study to propose an AI-driven therapeutic lifestyle change (AI-TLC) intervention. Through personalization, continuous tracking feedback, intelligent decision aid, evaluate the influence of AI-TLC intervention on health behavior and health outcomes of obese pregnant women, determine the applicability and universality of AI-TLC intervention, provide innovative, efficient, and personalized health intervention for obese pregnant women, and provide new perspectives and methods for related scientific research and clinical practice.

Specific objectives are:

Primary: Can the intervention program driven by artificial intelligence (AI)–facilitated therapeutic lifestyle changes effectively promote improvements in health behaviors and health outcomes among pregnant women who are overweight or obese?

Sub-question 1:

Do the effects of the intervention differ among pregnant women of varying ages and degrees of obesity?

Sub-question 2: From a cost-benefit perspective, which level of intervention is the most effective (human-only vs. AI-only vs. human + AI)?

Sub-question 3: What are the short-term, medium-term, and long-term effects of this intervention program? What mechanisms need to be added to achieve better long-term outcomes?

## 2 Methods and analysis

### 2.1 Study participants and sample size

**Figure 1:**
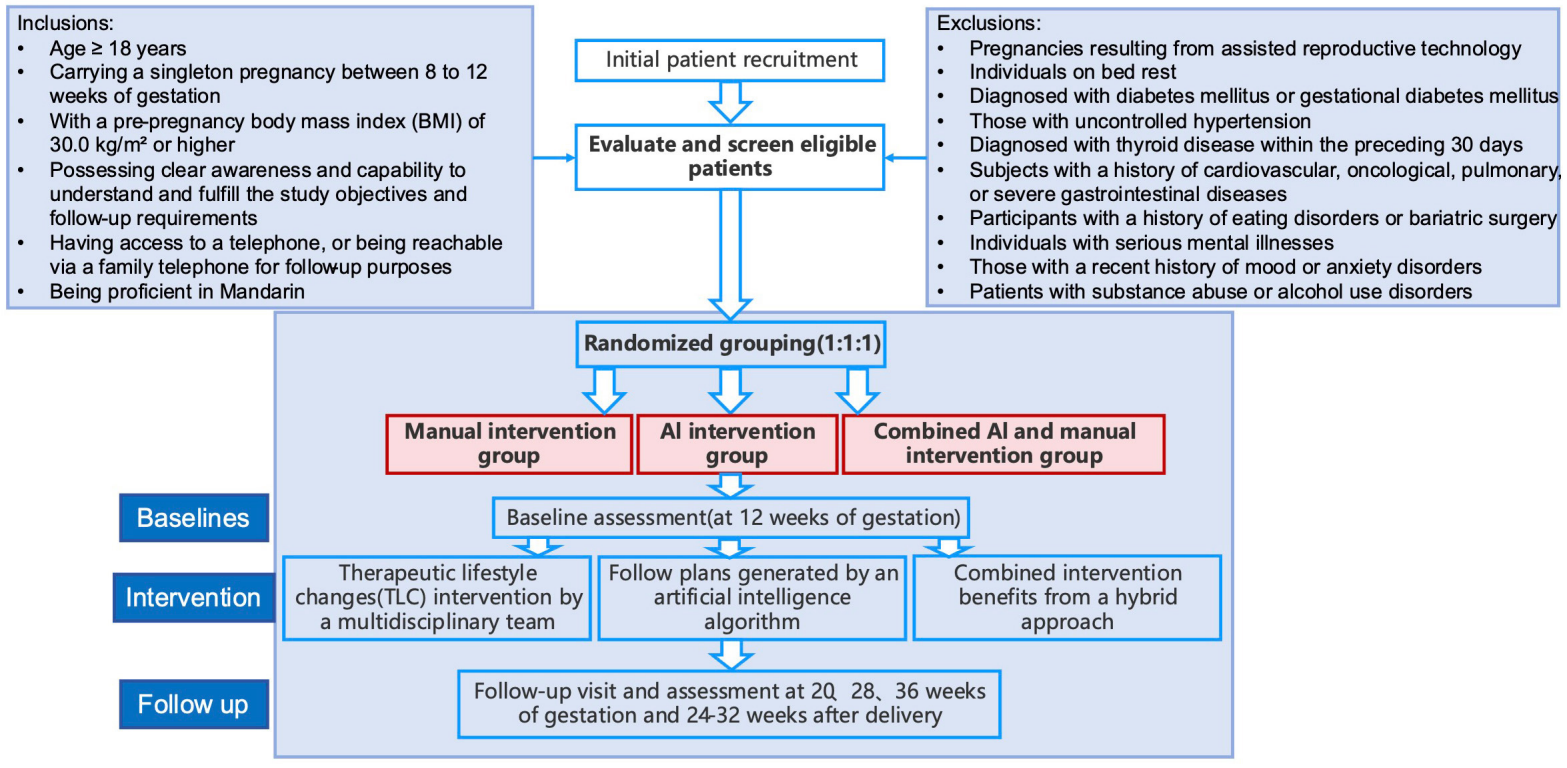
Flowchart of randomized controlled trial.

The baseline assessment for this study will be conducted at approximately 12 weeks of gestation. Follow-up visits are scheduled at approximately 20, 28, and 36 weeks of gestation, as well as at 3 months postpartum (24–32 weeks post-delivery). Figure 1 presents the detailed flowchart of this study.

#### 2.1.1 Sample size

As this study is a randomized controlled trial, the respondents will be divided into manual intervention group, artificial intelligence (AI) intervention group, artificial intelligence and manual combined intervention group, and observe the BMI of pregnant women, maternal weight and maternal adverse pregnancy outcomes as the main outcome indicators. The sample size was calculated based on an expected effect size of 0.5, which corresponds to the minimal clinically important difference (MCID) of the primary outcome. This MCID was determined based on previous literature and clinical judgment. Setting bilateral α = 0.05, the degree of certainty was 90%. Using the PASS15 software, we obtained a sample size of N1 = 40 cases for the group and N2 = 40 cases for the group and N3 = 40 cases for the group. For a total of at least 120 subjects to be included in the study.

#### 2.1.2 Recruitment

In this study, the selection of participants will follow the steps outlined as follows.

##### 2.1.2.1 Eligibility and inclusion criteria

In this study, pregnant women were initially identified within the electronic health record system based on the following eligibility criteria: aged 18 years or older; carrying a singleton pregnancy between 8 to 12 weeks of gestation; with a pre-pregnancy body mass index (BMI) of 28.0 kg/m^2^ or higher; possessing clear awareness and capability to understand and fulfill the study objectives and follow-up requirements; having access to a telephone, or being reachable via a family telephone for follow-up purposes; and being proficient in Mandarin, as subsequent interventions are conducted in Mandarin.

##### 2.1.2.2 Exclusion and withdrawal criteria

The following exclusion criteria were applied to the patient samples in this study: pregnancies resulting from assisted reproductive technology; individuals on bed rest; patients with a diagnosis of diabetes mellitus or gestational diabetes mellitus; those with uncontrolled hypertension; individuals with a recent diagnosis of thyroid disease within the preceding 30 days; subjects with a history of cardiovascular, oncological, pulmonary, or severe gastrointestinal diseases; participants with a history of eating disorders or bariatric surgery; individuals with serious mental illnesses; those with a recent history of mood or anxiety disorders; patients with substance abuse or alcohol use disorders; pregnancies beyond 13 weeks of gestation.

Additionally, the following samples were excluded from the study: participants who withdrew from the study; individuals experiencing severe acute diseases requiring treatment during the study period.

##### 2.1.2.3 Recruitment and grouping of participants

This study employed a multi-faceted recruitment strategy that integrated hospital-based and online channels to enroll pregnant women. Announcements regarding the clinical trial were disseminated via the WeChat official account of the Inner Mongolia Maternal and Child Health Hospital and associated collaborative medical institutions, with an electronic questionnaire QR code appended to the posts. Pregnant women and their families interested in participation were able to provide their contact details through the electronic questionnaire, after which they were contacted by study staff for further coordination.

Furthermore, outpatient surveys conducted at the Inner Mongolia Maternal and Child Health Hospital identified pregnant women presenting for check-ups or consultations with obesity-related symptoms. For those who expressed interest, a questionnaire survey was administered by the outpatient healthcare providers.

### 2.2 Randomization

After recruitment, the research team will conduct a pre-enrollment assessment and post-enrollment informed consent with the participants and their families, followed by randomization grouping:

A comprehensive assessment will be conducted before the participants are enrolled, including structured questionnaires and clinical examinations.

The approved subjects were randomly divided into Manual intervention group, artificial intelligence intervention group (AI intervention group), artificial intelligence and artificial combined intervention group (AI & Manual intervention group). The specific randomization operation is as follows: This study uses a simple randomization method. Random number series were randomly generated by the study designer using the RANDBETWEEN formula in EXCEL. The project will divide the subjects into different groups according to the sequence, the intervention team will know the grouping results, and the subjects will not know the grouping results.

### 2.3 Interventions

This study employs a therapeutic lifestyle changes (TLC) intervention to address the health behaviors of obesity pregnant women. The TLC strategy is comprehensive, encompassing three primary components: behavioral modification, dietary adjustments, and physical activity promotion, all facilitated by a multidisciplinary team comprising physicians, health managers, and nutritionists. Behavioral interventions involve advising patients to maintain daily records of weight, diet, and physical activity, to avoid prolonged sedentary behavior, to establish regular sleep patterns, to control eating pace, to ensure adequate hydration, and to reduce the frequency of dining out. Additionally, patients are encouraged to seek support from family and social networks and to engage in professional weight management education when required.

The dietary intervention is based on evidence-backed dietary patterns, including calorie restriction, very low-calorie diets, low-carbohydrate diets, high-protein diets, and intermittent fasting, all aimed at achieving a negative energy balance. Exercise guidance is tailored to the individual's health status and preferences, with recommendations developed under the supervision of qualified medical or fitness professionals. Psychological support is also integrated to bolster self-efficacy, mitigate stress, and alleviate symptoms of depression and anxiety, thereby enhancing the overall efficacy of the weight management program and improving the quality of life.

The fasting protocol in this study is structured as time-restricted eating (TRE), which limits food intake to an 8-h window each day, allowing for a 16-h fasting period. Participants are provided with detailed dietary guidelines that emphasize nutrient-dense foods, including adequate protein, healthy fats, and complex carbohydrates, to meet the increased nutritional needs during pregnancy. Regular monitoring of maternal weight gain and key health parameters, such as blood glucose and blood pressure, was conducted to ensure that fasting did not negatively impact maternal and fetal health. This helped in early detection and correction of potential nutritional deficiencies or excesses. Additionally, participants received education on healthy eating and lifestyle choices, emphasizing the selection of nutrient-dense foods during fasting and the avoidance of high-sugar, high-fat, and high-sodium foods to maintain a healthy weight and metabolic balance (26).

The research design incorporates a single-blind experimental setup, wherein participants are unaware of their allocation status or the identity of the intervention provider.

The Manual intervention group receives personalized TLC plans developed by the multidisciplinary team based on individual health profiles. The AI intervention group follows plans generated by an artificial intelligence algorithm using the same health data. The Combined intervention group benefits from a hybrid approach, where AI-generated plans are supplemented with expert recommendations from the healthcare professional team.

**Figure 2:**
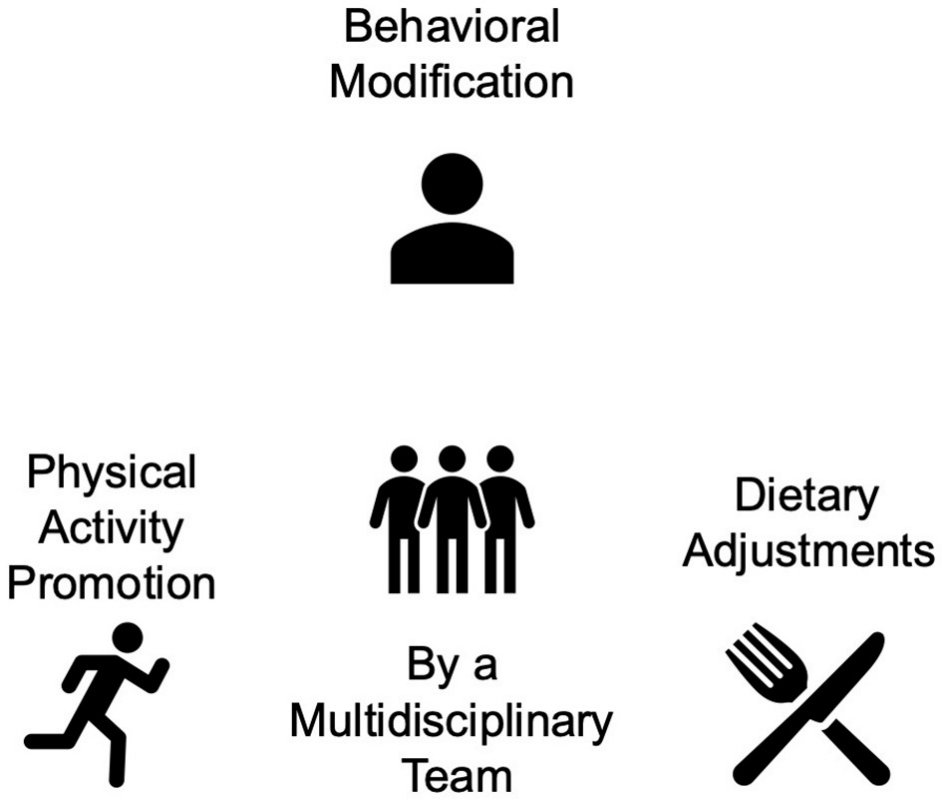
Therapeutic lifestyle changes (TLC) intervention.

This study's methodology aligns with the rigorous standards expected by academic journals, ensuring a controlled and systematic evaluation of the TLC intervention's impact on health behaviors and outcomes among obesity pregnant women (Figure 2).

### 2.4 Outcomes

#### 2.4.1 Primary outcomes

The primary outcomes of the study include maternal body mass index (BMI), maternal weight, and adverse pregnancy outcomes. The primary outcome measures (such as BMI, maternal weight, and adverse pregnancy outcomes) will be assessed using Repeated Measures ANOVA or Mixed Effects Models to evaluate the intervention effects. For long-term effect analysis, Generalized Estimating Equations (GEE) or Cox Proportional Hazards Models will be employed to assess the short-term, medium-term, and long-term impacts of the interventions. Cost-effectiveness analysis will be conducted using Quality-Adjusted Life Years (QALY) and Incremental Cost-Effectiveness Ratios (ICER) to evaluate the economic efficiency of different intervention measures, with sensitivity analysis performed to verify the robustness of the results.

#### 2.4.2 Secondary outcomes

The secondary outcomes encompass physiological indicators such as blood pressure and blood lipid profiles (including triglycerides, low-density lipoprotein (LDL), high-density lipoprotein (HDL), and very-low-density lipoprotein (VLDL) cholesterol levels), quality of life assessments, mental health evaluations, and lifestyle factors (dietary habits and physical exercise patterns). Secondary outcome measures (such as blood pressure, lipid levels, and quality of life) will be analyzed using multiple linear regression or logistic regression, controlling for confounding factors such as age and baseline BMI, to assess the independent effects of the interventions. Subgroup analysis will further explore the differences in intervention effects across subgroups defined by factors such as age and BMI classification, with interaction tests conducted to verify the robustness of the results.

### 2.5 Questionnaires

Health behaviors and disease prevention interventions in obesity pregnant women will be assessed using a general information questionnaire and specific scales, and mental health status will be measured by 7-item generalized anxiety disorder (GAD-7) (27), and the Self-Efficacy Scale, which reflect participants' anxiety, self-rated status, and stress levels over the past 2 weeks. Sleep quality will be assessed using the Pittsburgh sleep quality index (PSQI), including sleep quality, time to fall asleep, sleep duration, sleep efficiency, sleep disorders, hypnotic drugs, and daytime dysfunction. The level of physical activity will be assessed using the Physical Activity Rating Scale-3 (PARS-3). This scale evaluates the intensity, duration, and frequency of physical activity over the past month. The Family Health Scale short-form (FHS-SF) will be used to measure the social and family support component, which has high reliability and validity (28). The physical and mental health status of the obese comorbidity population will be based on the five-level five-dimensional health scale, and the World Health Organization's five physical and mental health indicators (WHO-5). The health status is judged by five dimensions: mobility, self-care, daily activities (such as work, study, housework, family or leisure activities), pain or discomfort, anxiety or depression, so as to propose targeted solutions to obesity-related factors. Health literacy will be assessed using Healthy Lifestyle Scale-Short Form 4 (HLS-SF4) that evaluates the ability to access, understand, and apply health-related information. Perceived stress will be measured using the Perceived Stress Scale (PSS), which assesses the frequency of feelings and thoughts related to stress over the past month. Social support will be evaluated using Perceived Social Support Scale that measures the perceived availability of emotional and practical support from family, friends, and significant others. The scale assesses the extent to which individuals feel cared for and supported in their personal and social lives (Table 1).

**Table 1:** Variables and measures collected at evaluation time points.

**Variables**		**Data source**	**Measures**	**Follow-up time point**					
				**T-0**	**T-1**	**T-2**	**T-3**	**T-4**	**T-5**
Covariates	Individual	Survey	Age, marital status, education level, occupation, annual income, ethnicity	√					
Social and demographic factors	Family related factors	Survey	Parents' medical history of hypertension, diabetes, and cardiovascular disease, annual family income	√					
Health status		Survey and Medical record	Height, weight, BMI, blood pressure and blood sugar, smoking and drinking status, WHO−5	√	√	√	√	√	√
Activity	Physical activities	Survey	Physical Activity Rating Scale-3, PARS-3	√	√	√	√	√	√
Psychosocial status	Quality of life	Survey	EQ-5D version, EQ-5D-5L	√	√	√	√	√	√
	Sleep	Survey	Pittsburgh sleep quality index, PSQI	√	√	√	√	√	√
	Social support	Survey	Social support rate scale, PSSS-SF	√	√	√	√	√	√
Psychology status	Anxiety	Survey	General Anxiety Disorder-7, GAD-7	√	√	√	√	√	√
	Stress	Survey	Perceived Stress Scale, PSS-4	√	√	√	√	√	√
	Self-efficacy	Survey	General Self-Efficacy Scale	√	√	√	√	√	√
	Family health	Survey	The Family Health Scale short-form, FHS-SF	√	√	√	√	√	√
Cost-effectiveness	Tracking cost	Survey and medical record		√	√	√	√	√	√
Qualitative results			Effect of trial	√	√	√	√	√	√

### 2.6 Statistical analysis

Participants' socio-demographics are presented as count and proportion, and the χ^2^ test was used to assess categorical variables. The continuous variables normally distributed were assessed by the student's *t*-test. The Fisher's exact or the chi-square test was adopted for categorical data, and statistical significance was set at *p* < 0.05. The main analysis was performed using generalized estimating equations (GEE) model. The GEE solved the problem of correlation of longitudinal data, utilizing the results of each measurement in the longitudinal data, which greatly reduces the loss of information (20). To clarify whether socioeconomic status affects the effectiveness of ILSM, we conducted several subgroup analyses for physiological health outcomes and health behaviors, and added the interaction terms to generalized estimating equations model to assess the interactions between time (at baseline, 3-month, 6-month, and 18-month) and group (intervention and control). We also used GEE to analyze the health benefits of intervention and control groups among participants with different socioeconomic status. Significance level of the associations in the final model was set at *P*-value 0.05. The data were analyzed using SPSS 20.0 (IBM Corporation, Armonk, NY, USA).

### 2.7 Study management

#### 2.7.1 Survey design phase

Prior to the official commencement of the study, a small-scale preliminary survey will be conducted. Based on the results of the preliminary survey and in conjunction with actual conditions, the recruitment and inclusion of research personnel will be refined, and the survey questionnaire will be perfected. Uniform professional training will be provided to all relevant research personnel to ensure the quality of the research work. This study follows the SPIRIT guidelines to ensure methodological rigor and transparency.

#### 2.7.2 Research implementation phase

The study will rigorously enforce the inclusion criteria for survey subjects, ensuring a meticulous selection and inclusion process into the study cohort. Participants with obesity pregnant women and their families will receive brochures detailing the prevention and treatment of chronic diseases, and informed consent will be obtained to ensure the authenticity of the feedback and to maintain a high response rate. Reminders will be sent via phone calls or text messages to minimize loss to follow-up during the survey process. Surveyors are committed to upholding principles of objectivity and equality, introducing the study's purpose and significance to each participant prior to commencing the survey and obtaining their informed consent. Furthermore, to ensure data integrity, the survey questionnaires will undergo regular review by auditors, with any inconsistencies or issues being addressed through immediate verification and correction.

#### 2.7.3 Data collection and analysis phase

In this study, we will utilize the “Wenjuanxing” (Questionnaire Star) online survey platform for questionnaire completion to ensure data accuracy. Before data analysis, we will conduct strict data organization and cleaning.

## 3 Discussion

The rising prevalence of obesity among Chinese pregnant women, as highlighted in the introduction (29). In a physical examination data based on 15.8 million adults, according to the Chinese standard (overweight BMI ≥ 24, obese BMI ≥ 28): the overweight rate is 34.8% and the obesity rate is 14.1%. Among the obese, 81.8% suffered from fatty liver, 36.9% had hypertension, and 42.4% had dyslipidemia (30). Consequently, there is an urgent need for healthcare interventions to both prevent and manage the development of obesity and its associated complications. Our study demonstrates that the AI-TLC intervention significantly improved maternal BMI and reduced adverse pregnancy outcomes compared to manual-only approaches. These findings align with prior research on technology-driven interventions. For instance, Ferrara et al. reported a 6-month BMI reduction of −0.9 ±1.5 in an mHealth-based intervention for gestational weight management (31), suggesting that digital tools can effectively support behavioral modifications. Similarly, our observation that the combined AI+Manual group outperformed standalone interventions echoes studies on hybrid models, where human-AI collaboration enhanced both adherence and personalization (23). Recent advances in AI interventions provide an appealing and cost-effective approach to intervention targeting the improvement of health outcomes for obese patients with other chronic disease comorbidity (30). To date, studies in Chinese obese patients with other chronic disease comorbidity have not been reported.

Our study introduces artificial intelligence driven therapeutic lifestyle changes (AI-TLC) intervention. The study aims to examine the effect of AI-TLC intervention on health behavior and health for obesity pregnant women. Several strengths warrant mentioning. Firstly, this study not only focuses on the treatment of obese individuals, but also considers obesity as a part of a pregnant women network and adopts multi-dimensional lifestyle modification as an intervention. This comprehensive intervention strategy is innovative in the management of obesity pregnant women. Secondly, an interdisciplinary team consisting of physicians, health managers, nutritionists, and artificial intelligence simulated health managers will be formed to provide comprehensive health management programs for obesity pregnant women. Thirdly, this study will introduce AI technology for health intervention to explore the application potential and effectiveness of AI intervention in obesity pregnant women management, which is prospective and innovative. Fourth, this study will conduct short-, medium- and long-term tracking and evaluation of the AI-TLC intervention effects, which will help to comprehensively understand the long-term impacts of the AI-TLC interventions and provide a basis for the development of sustained and effective health management strategies. Fifth, this study will evaluate the economic benefits of different interventions from a cost-benefit perspective and help to provide policymakers and healthcare providers with cost-effective intervention options.

Here are some extended comparisons with similar studies. The efficacy of AI-TLC interventions can be further contextualized by contrasting it with other obesity management strategies. Recent systematic reviews highlight that AI-driven interventions excel in providing real-time personalized feedback, a feature critical for sustaining behavioral changes in high-risk populations (32). For example, hybrid interventions combining digital tools and human support have shown superior long-term weight maintenance compared to purely automated systems, supporting our finding that the AI+Manual group achieved the highest clinical impact (33). This aligns with broader evidence that technology-enhanced programs improve accessibility in resource-limited settings (34), a key consideration for China's maternal healthcare system.

Next are the differences with the existing literature. Notably, our cost-benefit analysis revealed that the AI-only intervention offered a scalable solution with moderate efficacy, whereas the combined AI+Manual group achieved the highest clinical impact. This contrasts with Perdomo et al., who emphasized surgical and pharmacological interventions as primary strategies for severe obesity (15). However, our results align with Sallam et al., where TLC combined with medication synergistically improved metabolic outcomes (20). Such parallels suggest that hybrid models (AI + human expertise) may bridge the gap between resource-intensive and purely automated interventions.

However, this study has the following limitations. First, despite the high coverage rate of smartphones in China, some patients may not be able to use them well, which may hinder the implementation and reduce the effectiveness of AI interventions. Second, for the collection of the primary and secondary outcomes, we primarily used questionnaire indicators, which may inevitably lead to recall bias. Third, due to the lack of balanced stratification, comparative analyses between subgroups may not be suitable if the sample size of each group is insufficient across groups (e.g., compare the differences and potential influencing factors of AI-TLC intervention effects in different age groups). Fourth, the short-term follow-up period (up to 3 months postpartum) precludes conclusions about long-term weight maintenance. Meta-analyses of lifestyle interventions for obese pregnant women indicate that postpartum weight retention remains a challenge beyond 12 months (35), emphasizing the need for extended follow-up in future studies. Additionally, socioeconomic disparities in AI accessibility—particularly among older or rural populations—may limit generalizability, necessitating stratified analyses in broader cohorts.

Despite these limitations, this study may provide a scientific basis for developing personalized and effective interventions to further improve the health behavior and health of obesity pregnant women. It is expected to provide a new perspective and effective approach to the management of obesity pregnant women.
